# Nonfunction of the ECT2 gene may cause renal tubulointerstitial injury leading to focal segmental glomerulosclerosis

**DOI:** 10.1007/s10157-012-0636-0

**Published:** 2012-05-03

**Authors:** Akane Izu, Keisuke Sugimoto, Shinsuke Fujita, Hitomi Nishi, Yutaka Takemura, Mitsuru Okada, Tsukasa Takemura

**Affiliations:** Department of Pediatrics, Kinki University School of Medicine, 377-2 Ohno-higashi, Osaka-Sayama, 589-8511 Japan

**Keywords:** Hyperfiltration, Tight junction, Tubular epithelial cell, Tubulointerstitial injury

## Abstract

**Background:**

Secondary focal segmental glomerulosclerosis (FSGS) follows congenital or acquired tubulointerstitial alterations such as in Dent’s disease, Lowe syndrome, and reflux nephropathy. Failure of adequate regeneration after tubulointerstitial injury, or abnormal tubulogenesis, can disturb intrarenal blood circulation, causing excessive glomerular filtration. The epithelial cell-transforming sequence 2 gene (*ECT2*) contributes to tight junction function in epithelial cells.

**Methods:**

We encountered two patients with a nonfunctioning *ECT2* genotype who later developed FSGS. Both developed proteinuria associated with acute renal failure in early childhood.

**Results:**

Renal biopsy specimens showed marked tubulointerstitial nephritis at the onset of proteinuria, later progressing to FSGS consequent to tubulointerstitial injury. The patients did not respond to corticosteroids and attained only incomplete remission upon cyclosporine A administration. One patient received a maternal renal transplant with good function and no rejection.

**Conclusions:**

*ECT2* is important for tight junction function and maintenance of cell polarity. Nonfunction of this gene may cause renal tubulointerstitial injury, progressing to glomerular sclerosis.

## Introduction

Focal segmental glomerulosclerosis (FSGS) may present with rapid development of systemic edema, often manifesting nephrotic syndrome (NS), microscopic hematuria, and hypertension [1]. In children, FSGS may be diagnosed after detection of proteinuria or hematuria on screening examination in a health and developmental check-up, sometimes progressing slowly. While FSGS can occur over a wide range, it frequently develops in children and young adults, sometimes progressing to end-stage renal failure [1].

FSGS includes primary and secondary forms. In primary FSGS, abnormality of genes encoding proteins constituting the slit membrane, which is responsible for the filtration function of glomerular epithelial cells, has been reported; glomerular epithelial cell impairment thus has been implicated [2]. However, no abnormality in these genes was observed in many patients with FSGS. Secondary FSGS occurs when glomerular epithelial cells are impaired by drugs or infection, and also in diseases with reduced numbers of nephrons such as congenital renal dysplasia. Hyperfiltration-induced abnormalities in renal circulatory dynamics then impair glomerular epithelial cells [1, 2]. Secondary glomerulosclerosis also develops from congenital or acquired uriniferous tubulointerstitial disorders such as Dent’s disease, Lowe syndrome, and reflux nephropathy [3–5].

Histopathologically, early lesions arise in the corticomedullary junction, and focal sclerosis is observed in the loops of less than 80 % of all glomeruli. FSGS variants have been classified into peripheral, cellular, tip, and collapsing types [2]. Despite the glomerular lesion of the primary lesion of FSGS, tubulointerstitial lesions and arteriolar hyalinization appear early in some patients; these lesions are important in the progression to renal failure [1–3].

The product of the epithelial cell transforming sequence 2 (*ECT2*) gene is a transforming protein related to Rho-specific exchange factors and cell-cycle regulators [6]. ECT2 protein is present at cell-to-cell contact sites and in the nucleus; it is involved in cell polarity, organogenesis, and structure and function of intercellular tight junctions [7].

We encountered two patients with intractable nephrotic syndrome in whom acute renal failure developed, both with severe tubulointerstitial disorders, followed by FSGS lesions. A nonfunctioning genotype of the *ECT2* was noted in these patients, suggesting an ECT protein deficiency in uriniferous tubular epithelial cells causing tubulointerstitial disorder, followed by development of FSGS lesions resulting from abnormal renal circulatory dynamics. This sequence of changes is informative with regard to the development of tubulointerstitial lesion-associated FSGS.

## Subjects and methods

### Subject

**Fig. 1:**
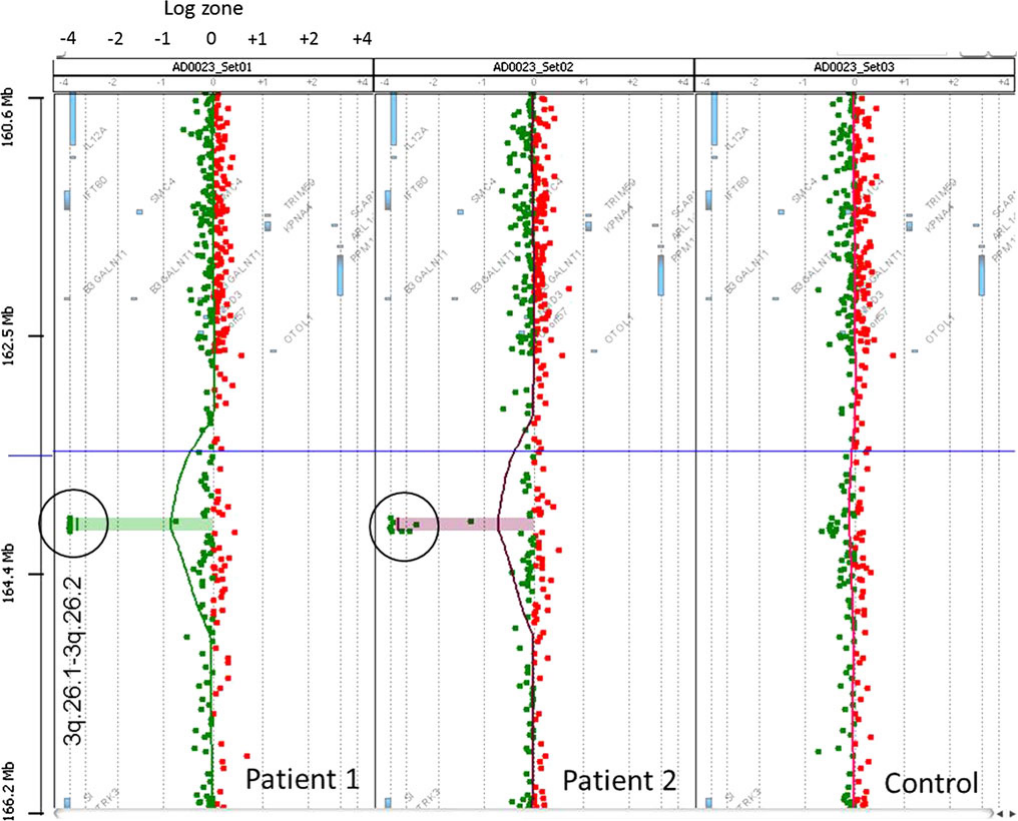
CGH findings in two patients and another FSGS patient. In the two patients described here, some clustered genes localized in chromosome 3q.26.1–3q.26.2 showed downregulation. Signal indicating the loss of copy number was recognized in the log4 zone, suggesting homozygous deletion of *ECT2* in both patients

Gene expression was screened by the comparative genomic hybridization (CGH) in 15 FSGS patients under treatment at our department [8]. In one patient, α-actinin 4, located on chromosome 19q.13, was deleted. In another, a 6p deletion-associated E2F3 gene aberration was found [9]. No abnormality was noted in α-actinin 4, nephrin (located at 19q13.1 and responsible for forming the slit membrane of glomerular epithelial cells), or in the CD2-associated protein gene (*CD2AP*, located at 6p12) in the remaining 13 patients. However, downregulation of *ECT2*, located at 3q26.1 to q26.2, was observed in two patients (Fig. 1). Thus, clinical and histological features were investigated in these patients to examine the association between *ECT2* and FSGS.

### Methods

#### Comparative genomic hybridization method

Array-CGH was used to screen for genes showing up- or downregulation in each subject. We obtained genomic DNA from a reference sample (46,XY) (Promega p/n G1471) and the present patients. CGH was performed using prefabricated oligo-CGH arrays (244-kb arrays; Agilent Technologies, Palo Alto, CA, USA) consisting of about 244,000 in situ-synthesized 60-mer oligonucleotides spanning the entire genome, resulting in an average genomic distance of approximately 12 kb. These probes included both coding and noncoding sequences on every human chromosome. After hybridization had been carried out according to the manufacturer’s instructions, results were visualized using CGHAnalytics 3.4 software (Agilent Technologies).

#### Polymerase chain reaction

Genomic DNA was recovered in the aqueous phase and precipitated with ethanol/sodium acetate. The polymerase chain reactions (PCR) were carried out as described previously [9]. Specific primers were constructed based on previously published sequence data for human *ECT2* coding regions [7]. PCR conditions were as follows: initial denaturation at 94 °C for 5 min, followed by 35 cycles of denaturation at 94 °C for 30 s, annealing at 63 °C for 30 s, and extension at 72 °C for 4 min. Analysis of* ECT2* was performed after we obtained written informed consent from the patients’ parents or guardians.

#### Immunohistochemical staining

Anti-ECT2 antibody was purchased from Santa Cruz Biotechnology (Santa Cruz, CA, USA). Staining for ECT2 protein in renal tissues was carried out using a previously described immunofluorescence method [9].

## Patient presentation

### Patient 1

**Fig. 2:**
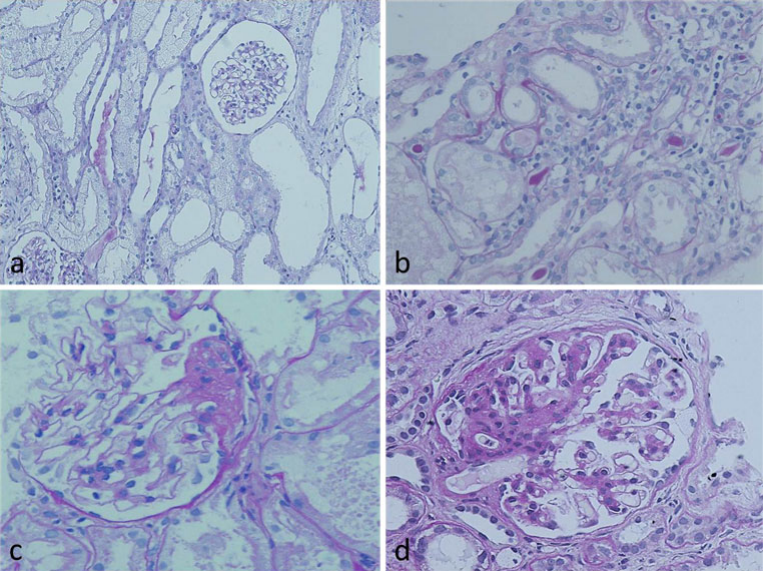
Histologic findings in patient 1. On initial biopsy at 3 years of age, tubulointerstitial alterations included tubular cloudy degeneration, cystic dilatation of tubules, detachment of tubular epithelial cells, and interstitial mononuclear cell infiltration (**a**, **b**); however, glomeruli were essentially normal. At the time of the second biopsy, focal segmental sclerosis of glomeruli was observed (**c**). These sclerotic lesions progressed together with tubulointerstitial changes in a specimen at age 8 (**d**)

The patient is a boy who is currently 8 years old. No abnormality had been noted in the perinatal period, and he was born by spontaneous delivery at full term. He is an only child and has no siblings. His parents were unrelated and healthy. No inherited kidney disease or other congenital anomalies of the kidney were found in his family members. At 3 years of age, he was brought to our department because of facial edema developing after acute enteritis. No contributory family or past medical history was obtained. On admission, systemic edema and ascites were evident. Mild mental retardation was present (Wisconsin Intelligence Scale for Children or WISC: 70), but motor functions were normal. Urinary protein was 4+ (8.7 g/day). In serum, total protein was 4.4 g/dl, and albumin was 2.1 g/dl, indicating NS. Blood urea nitrogen (BUN) was 59 mg/dl and creatinine was 1.23 l, showing renal hypofunction. Urinary β2-microglobulin (MG) was increased by 1,450 μg/day; however, the urine concentrating ability, osmotic pressure of the urine, and excretion of several minerals into the urine were normal. Steroid therapy (2 mg/kg/day) was initiated, but urinary protein did not decrease. A renal biopsy specimen included 16 glomeruli; changes were minimal (Fig. 2a). However, marked cloudy degeneration and vacuolation of uriniferous tubules and tubular epithelial cell detachment were noted, and the uriniferous tubules showed cystic changes (Fig. 2a, b). Immunofluorescence methods showed no deposition of any immunoglobulin type or of complement. Localization of nephrin and CD2AP was normal. The patient was diagnosed with steroid-resistant NS. Cyclosporin A (CyA) treatment was initiated, obtaining a type I incomplete remission. At 4 years of age, proteinuria was exacerbated by infection, and the patient was admitted for treatment. In a second kidney biopsy specimen, segmental sclerotic glomerular lesions were observed, leading to the diagnosis of FSGS (Fig. 2c). In a third biopsy specimen at 6 years of age, tubulointerstitial and segmental sclerotic glomerular lesions had progressed (Fig. 2d). In the specimen obtained at 4 years, the median diameter was 92.4 μm in 32 glomeruli evaluated, representing about 1.5 times that seen in age-matched children (55–60 μm); the number of glomeruli per unit area was 5.2/mm^2^, a value within the normal range. The number of glomeruli had decreased and glomerular diameter increased in the subsequent specimen. No non-functioning genotype of *ECT2* was observed in his parents, suggesting a de novo case.

### Patient 2

**Fig. 3:**
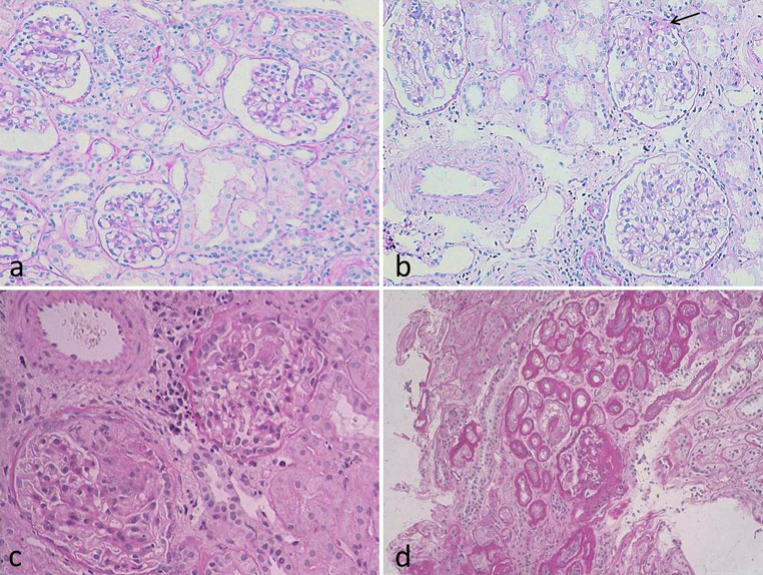
Histological findings in patient 2. On initial biopsy at 3 years of age, tubulointerstitial alterations including detachment of tubular epithelial cells, atrophic changes of renal tubular membranes, and interstitial edema were present (**a**, **b**); however, glomeruli were normal. A second biopsy specimen obtained at 5 years showed focal segmental sclerosis of glomeruli (**c**). These sclerotic lesions progressed together with tubulointerstitial changes in a specimen at age of 8 (**d**)

The patient is a man who is currently 24 years old. No abnormality had been noted in the perinatal period, nor was there any contributory or past medical history. His parents were unrelated; however, they were divorced soon after his birth. No inherited kidney disease or other congenital anomalies of the kidney were found in his maternal family members. The patient was brought to our department because of edema that developed after influenza at 3 years of age. Proteinuria, hypoproteinemia, and mild renal dysfunction were present, and the patient was admitted. On physical examination, facial edema was present, but ascites was absent. Mild mental retardation was noted, but motor function was normal. Semiqualitative urinary protein was 4+ (5.4 g/day). Serum total protein was 4.2 g/dl, and albumin was 2.1 g/dl, indicative of NS. BUN was 33 mg/dl and creatinine was 1.4 mg/dl, showing mild renal hypofunction. Urinary β2-MG was 1,020 μg/day, representing a mild increase; however, the urine concentrating ability remained normal at this time. Steroid therapy (2 mg/kg/day) was initiated, but urinary protein levels did not decrease. Kidney biopsy was performed, obtaining 23 glomeruli; changes were minimal. In the uriniferous tubular interstitium, tubular epithelial cell detachment, focal thickening and atrophy of the tubular basement membrane, and mild interstitial fibrosis were observed (Fig. 3a). Immunofluorescence showed no deposition of any immunoglobulin type or of complement. Localization of nephrin and CD2AP was normal. The patient was diagnosed with steroid-resistant NS. CyA treatment was initiated, obtaining a type I incomplete remission. A second kidney biopsy was performed at 5 years of age because of increased proteinuria. Glomerular enlargement had progressed, and segmental sclerotic lesions were noted in some glomeruli. Based on the later findings, FSGS was diagnosed (Fig. 3b, arrow). In a third specimen at 8 years of age, tubular atrophy, tubular interstitial fibrosis, and glomerular segmental sclerotic lesions had progressed (Fig. 3c, d). The median glomerular diameter was 73.5 μm in the specimen obtained at 5 years (25 glomeruli evaluated), slightly larger than in age-matched children (55–60 μm); the number of glomeruli per unit area was 5.8/mm^2^, within the normal range. However, in the next specimen, the number of glomeruli had decreased (4.7/mm^2^) and glomerular diameter increased. Since we were not able to obtain consent for gene analysis from his mother, the mode of inheritance remains unclear.

## Immunohistologic and genetic examination in these patients

**Fig. 4:**
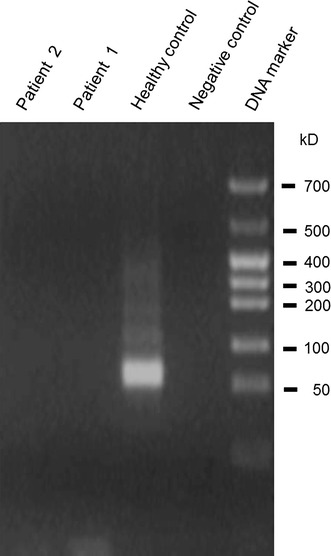
Polymerase chain reaction for *ECT2*. To confirm deletions, PCR was carried out by using primers specific for human *ECT2* based on previously published sequence data. In patients 1 and 2, no amplification band was detected, confirming the CGH results

To confirm *ECT2* deletions, PCR for *ECT2* was carried out. In patients 1 and 2, no amplification band was detected (Fig. 4), confirming the CGH results. In the remaining 13 patients with FSGS examined and the additional 50 healthy volunteers, no non-functioning genotype of *ECT2* was demonstrated except for each of three independent silence mutations of this gene having no amino acid substitution in the three individuals (2 are healthy volunteers and 1 is FSGS patient). Although there is no database concerning the incidence of mutation or deletion in *ECT2* at present, at least in the 63 samples examined in this study, cases with homozygous deletion of *ECT2* were not found.

**Fig. 5:**
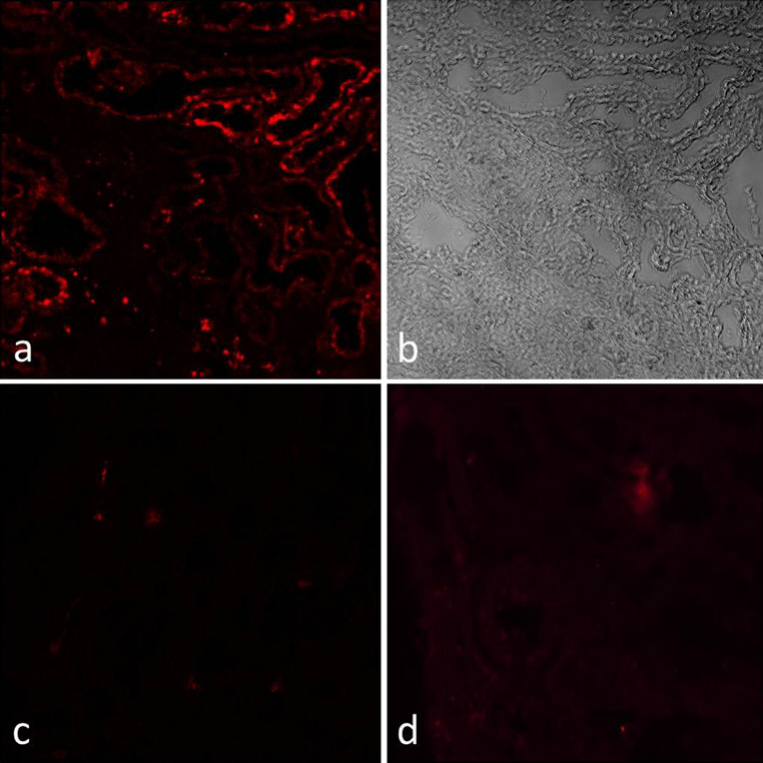
ECT2 protein expression in renal specimens from our patients compared with a normal renal specimen by immunofluorescence using anti-ECT2 antibody. Histologically normal portions of specimens obtained from patients with renal trauma served as normal kidney tissue. In the normal kidney specimen, ECT2 protein was localized in the renal tubules (**a**), which was confirmed by phase-contrast microscopy (**b**), while in the two patients, expression was absent at these sites (**c** patient 1, **d** patient 2)

Immunohistological evaluation for ECT2 protein in these two patients revealed no expression of this molecule in renal tubular epithelium (Fig. 5).

## Discussion

FSGS includes primary and secondary forms. In primary FSGS, aberrant *CD2AP* and Wilms' antioncogene (*WT1*), which encode proteins constituting the slit membrane responsible for the filtration function of glomerular epithelial cells, have been reported, suggesting glomerular epithelial cell impairment [1, 2, 9]. Familial or hereditary development of FSGS has also been reported in association with gene aberration of inverted formin 2, *ACTN4*, and *MYH9* [10–13]. However, no abnormality was noted in these reported genes in many patients with FSGS. Secondary FSGS may occur when glomerular epithelial cells are impaired by drugs such as heroin, HIV infection, or conditions with reduced numbers of nephrons such as congenital renal disease, low birth weight, oligomeganephronia, and renal dysplasia [1, 3]. Reduction in the number of nephrons can cause hyperfiltration-induced renal circulatory dynamics abnormalities that impair glomerular epithelial cells. Secondary glomerulosclerosis also develops from congenital or acquired renal tubulointerstitial disorders such as Dent’s disease, Lowe syndrome, and reflux nephropathy, an important causative disease of terminal renal failure; FSGS lesions have been observed in the course of these diseases [2, 3].

Tight junctions function as an intercellular barrier regulating paracellular permeability in vertebrate epithelial and endothelial cells [14]. They also provide physical “fences” within the membrane bilayer that prevent intermixing of membrane proteins, thus maintaining cell surface asymmetry. Furthermore, they provide essential structures and serve as specific sites for vesicle targeting to establish and maintain epithelial polarity of the cell membrane [14]. Tight junctions are composed of large complexes of cytoplasmic and membrane proteins. Adapters such as tight junction protein ZO-1 and signaling molecules such as small GTPases are components of the complexes [15]. Additionally, Par6, Par3, and atypical protein kinase C (aPKC) localize to tight junctions in Madin-Darby canine kidney (MDCK) cells [16]. Activation of Par6 or overexpression of aPKC regulates formation of tight junctions. On the other hand, cell polarity regulates diverse biological events such as localization of embryonic determinants and establishment of tissue and organ architecture [17]. Epithelial cell polarity is known to be regulated by the polarity complex Par6/Par3/aPKC [15].

Polarized epithelial cells maintain an asymmetric composition of their apical and basolateral membrane domains by at least two different processes [18]. These include regulated trafficking of macromolecules from the biosynthetic and endocytic pathway to the appropriate membrane domain and prevention of free mixing of membrane domain-specific proteins and lipids by the tight junction. Cdc42, a Rho family GTPase, is known to govern cellular polarity and membrane traffic in several cell types [19, 20]. Expression of dominant-active Cdc42V12 or dominant-negative Cdc42N17 in MDCK cells was found to alter tight junction function, indicating that Cdc42 may modulate the multiple cellular pathways required for maintenance of epithelial cell polarity [20]. Nucleotide exchange factor *ECT2* stimulates guanine nucleotide exchange on RhoA, Rac1, or Cdc42 in vitro [21]. Another study disclosed that ECT2 also associates with this polarity-related complex and regulates aPKC activity. MDCK cells expressing a dominant-negative form of ECT2 are unable to form normal cystic structures with central lumens in three-dimensional collagen gels [22]. Thus, lack of ECT2 molecules in renal epithelial cells could disturb normal development in organs including renal tubulogenesis as well as regeneration of renal tubules after injury. However, since genetically engineered animals lacking *ECT2* have not been established, the crucial role of *ECT2* for renal tubular function or architecture except for tight junction function remains uncertain.

Even before the appearance of glomerular lesions, FSGS shows greater glomerular diameters than does minimal change nephrotic syndrome (MCNS). Also, a tubulointerstitial disorder develops early in FSGS, but generally does not develop in MCNS [22]. In our patients, the number of glomeruli per unit area was normal in early specimens, but glomerular diameter was greater than in age-matched normal specimens. Glomerular enlargement progressed and the number of glomeruli decreased together with the progression of tubulointerstitial lesions in later biopsy specimens. Possibly, deletion of *ECT2*, which is essential for embryonic development and maintenance of the function of uriniferous tubules, caused tubular dysplasia, and when the tubulointerstitial disorder progressed postnatally after an infection, the renal circulation was disturbed. As the number of glomeruli decreased, hyperfiltration by residual glomeruli induced FSGS lesions [23]. Patient 1, now 8 years old, had a severe tubulointerstitial disorder from the time of the first episode. Renal function slowly declined, with the current creatinine clearance declining to 62.5 ml/min. Patient 2, now 24 years old, had a tubulointerstitial disorder progressing after clinical presentation at age 3; glomerulosclerotic lesions were present at 5 years. The condition progressed to end-stage renal failure at 14 years of age. He received a kidney transplant from his mother, and a favorable outcome was achieved. Both patients improved with immunosuppression to show type I incomplete remission, but progression of renal failure could not be prevented.

Since many molecules including ECT2 participate in tight junction function, we assumed that the structure and function of uriniferous tubules were essentially intact initially, even though the ECT2 protein was deficient. Later, secondary glomerulosclerosis followed destruction of the tubular architecture, and renal failure reached the end stage as the number of glomeruli decreased. Both patients were unresponsive to steroids because the disease developed from *ECT2* deletion, not through autoimmunity. Recurrence after renal transplant was not seen in patient 2. Mild mental retardation was noted in both patients, but a causal relationship to the *ECT2* deletion is unclear.

We encountered two FSGS patients with a non-functioning genotype of *ECT2*. The result was deficiency of a protein that maintains uriniferous tubular polarity and function of tight junctions. As the pathogenesis of FSGS is heterogeneous, these patients are interesting with regard to their FSGS apparently complicating tubulointerstitial lesions. However, precise mechanisms for renal tubular dysfunction caused by the non-functioning genotype of *ECT2* were not fully addressed in this study; thus, the determination of the direct role of this gene for renal tubules using functional analysis would be necessary in future studies.
